# MBOVPG45_0375 Encodes an IgG-Binding Protein and MBOVPG45_0376 Encodes an IgG-Cleaving Protein in *Mycoplasma bovis*

**DOI:** 10.3389/fvets.2021.644224

**Published:** 2021-04-15

**Authors:** Haoran Zhao, Yunke Zhang, Zhanhui Wang, Mengyao Liu, Pengpeng Wang, Wenxue Wu, Chen Peng

**Affiliations:** Key Laboratory of Animal Epidemiology and Zoonosis, College of Veterinary Medicine, China Agricultural University, Beijing, China

**Keywords:** *Mycoplasma bovis*, MBOVPG45_0375, MBOVPG45_0376, IgG-binding protein, IgG-cleaving protein, membrane protein

## Abstract

*Mycoplasma bovis* is a significant bacterial pathogen which is able to persist in cattle and cause chronic diseases. This phenomenon may relate to *M. bovis* evading the immune system of the host. Immunoglobulin-binding proteins are widely distributed in a variety of pathogenic bacteria, including some *Mycoplasma* species. These proteins are considered to help the bacteria evade the immune response of the host. Here we found *M. bovis* strain PG45 can bind to IgG from several animals. MBOVPG45_0375 encodes a putative membrane protein, has strong amino acid sequence similarity with Immunoglobulin G-binding protein in *Mycoplasma mycoides* subsp. *capri*. Hence, we constructed recombinant MBOVPG45_0375 (r0375) in the *Escherichia coli* expression system and demonstrated that r0375 can bind to IgG non-immunologically rather than specific binding similar to interaction of antigen and antibody. Moreover, r0375 can bind to the Fab fragment of IgG. Also, the binding of r0375 and IgG inhibits the formation of antigen-antibody union. Furthermore, MBOVPG45_0376 encodes an IgG-cleaving protein of *M. bovis* strain PG45. Nevertheless, r0375 binding to IgG is required for the cleavage activity of recombinant 0376 (r0376). The activity of r0376 is also affected by incubation time and temperature. In addition, we found both MBOVPG45_0375 and MBOVPG45_0376 are membrane proteins of *M. bovis* strain PG45. These results about MBOVPG45_0375 as an IgG-binding protein and MBOVPG45_0376 as an IgG-cleaving protein offer a new insight into the interaction between *M. bovis* and its host.

## Introduction

Mycoplasmas are considered as a special class of bacteria as they are minimal-size pathogenic bacteria and lack a cell wall (1). Although the genome of mycoplasmas is not as complex as most bacteria, they can infect humans and a variety of animals (2). *Mycoplasma bovis* is a significant bacterial pathogen that is able to persist in cattle and cause chronic diseases, such as pneumonia, mastitis, otitis, reproductive disorders, arthritis, meningitis, and corneal conjunctivitis (3–5). On cattle farms, the morbidity of *M. bovis* is relatively high. Though the mortality rate is low, economic losses caused are considerable (6). The occurrence of these chronic infections suggests that *M. bovis* has strategies to escape host immunity. Currently, the findings related to *M. bovis* immune evasion mainly includes a high frequency variation of variable membrane surface lipoproteins (Vsps) (7), avoiding phagocytosis of neutrophil granulocytes and macrophages (8), and modulating immune responses of the host (1).

Up to the present, only a few virulence factors of mycoplasmas have been identified and researched extensively. For instance, the community acquired respiratory distress syndrome toxin of *Mycoplasma pneumoniae* has vacuolating activity (9). *Mycoplasma arthritidis*'s mitogen, an immunomodulatory protein, can induce excessive immune reaction (10). In addition, some immunoglobulin-binding proteins (IBP) were found in multiple mycoplasma species (11–14). IBP are widely distributed in a variety of pathogenic bacteria. These proteins are considered to help the bacteria evade the immune response of the host by allowing immunoglobulins to cover the bacterial cell surface and forming camouflage. Well-characterized examples include protein A of *Staphylococcus aureus* (15), protein G of group C and G streptococci (16), and protein L of *Peptostreptococcus magnus* (17). Protein A and G primarily bind to the Fc domain of IgG, but protein L binds to the Fab domain of IgG. In mycoplasmas, *Mycoplasma salivarium* contains a cellular protein that can bind the Fc fragment of human IgG (11). Both *Mycoplasma hominis* and *Mycoplasma arginini* possess a 95–105 kDa protein which reacts with the Fab fragments of IgG from several animals (12). Protein M in *Mycoplasma genitalium* and mycoplasma immunoglobulins binding protein (MIB) in *Mycoplasma mycoides* subsp. *capri* were found in recent years, and both these proteins can bind to the Fab domain of IgG (13, 14). Protein M and MIB have a strong structural similarity despite very limited sequence similarity between them (14). Importantly, homologs of protein M and MIB are distributed in various mycoplasma species (14).

The MIB-MIP in *M. mycoides* subsp. *capri* is a mycoplasma system that captures and cleaves IgG. The gene of immunoglobulins cleaving protein (MIP) is in tandem with the gene of MIB, and MIP is a serine protease that can proteolytically cleave off the variable region of IgG heavy chains (14). In addition, some immunoglobulin-cleaving proteins are also found in *M. synoviae, M. gallisepticum*, and *Ureaplasma* (18–20). Although these proteases have different substrates and cleavage sites, they probably have a common function that helps mycoplasma to avoid the host immunity. Interestingly, MIB and MIP homologs are found in the majority of animal pathogenic mycoplasmas, including *M. bovis* (14, 21). Furthermore, there are multiple paralogs that are present in most *M. bovis* genomes. However, the predicted activity of MIB and MIP homologs in *M. bovis* requires further assessment.

In this study, we focused on identifying the IgG-binding proteins and IgG-cleaving proteins of *M. bovis*. We have identified that *M. bovis* strain PG45 can bind IgG from several animal species, which confirms that *M. bovis* contains IBP as predicted from previous studies (14, 21). After analyzing the genome information of *M. bovis* strain PG45, we found MBOVPG45_0372, 0374, and 0375 encode putative membrane proteins that have high amino acid sequence similarity with MIB, MBOVPG45_0373, and 0376 encode putative lipoproteins that have high amino acid sequence similarity with MIP. According to the amount of recombinant protein expressed in *Escherichia coli*, we chose MBOVPG45_0375 and MBOVPG45_0376 as representatives to identify the function of these paralogs. We have confirmed r0375 has the function of binding IgG non-immunologically, and r0375 binds to the Fab domain of IgG. Union of antigen-antibody and deposition of C1q on *M. bovis* strain PD mediated by IgG are partly blocked by r0375. Furthermore, we found that r0376 can cleave IgG based on the combination of r0375 and IgG. The activity of r0376 varied under different incubation time and temperature. These data confirm that r0376 is a protease. In addition, we have confirmed both MBOVPG45_0375 and MBOVPG45_0376 are located on the membrane. These results of r0375 and r0376 indicate that they may be related to the immune evasion of *M. bovis*.

## Materials and Methods

### Ethics Statements

All animal studies were performed in accordance with the China Agricultural University Institutional Animal Care and Use Committee guidelines (CAU20180401-2) and followed the International Guiding Principles for Biomedical Research Involving Animals. Experiments were approved by the Beijing Administration Committee of Laboratory Animals.

### Reagents and Antibodies

Bovine IgG powder and Coomassie brilliant blue were purchased from the Solarbio Company (Solarbio, Beijing, China). polyvinylidene fluoride (PVDF) membrane was purchased from Millipore Corporation (Billerica, MA, USA). Restriction enzymes *Bam*H I and *Xho* I were purchased from the New England BioLabs Company (Ipswich, MA, USA). Fab and Fc fragments of human IgG were purchased from Abcam (Cambridge, UK). HRP-conjugated goat anti-rabbit antibody and goat anti-mouse antibody were purchased from Santa Cruz Biotechnology (Dallas, TX, USA). HRP-conjugated rabbit anti-bovine IgG and rabbit anti-horse IgG were purchased from the Solarbio Company. HRP-conjugated goat anti-swine IgG and rabbit anti-chicken IgG (IgY) were purchased from the ABclonal Company (ABclonal, Wuhan, China). All chemical reagents used in the study are analytical grade.

### Bacteria Strains and Culture Conditions

*Mycoplasma bovis* strain PG45 (ATCC® 25523TM, acquired from the China Veterinary Culture Collection Center, Beijing, China) and *M. bovis* strain PD (Laboratory culture collection) were grown in modified PPLO medium containing 20% horse serum at 37°C in 5% CO_2_ as described previously (22). *Escherichia coli* BL21 (DE3) (TransGen Biotech, Beijing, China) was propagated at 37°C in Luria–Bertani (LB) broth or on LB agar plates with 50 μg kanamycin/mL when selection was needed.

### ELISA Assay to Identify Whether *Mycoplasma bovis* Strain PG45 Binds IgG

Microtiter polyvinyl plates (Corning, NY, USA) were coated with different concentrations of *M. bovis* and incubated overnight at 4°C. The plate were washed three times with PBST (PBS, 0.05% Tween 20, PBST). Then the plates were blocked with blocking buffer [5% (wt/vol) skim milk (BD, Sparks, MD, USA) in PBST] for 2 h at 37°C. After washing, the plates were incubated with non-specific IgG from cattle, horse, rabbit, swine, mouse, and chicken (Solarbio, Beijing, China) for 1 h at 37°C. Recombinant P46 (rP46, Laboratory collection) of *M. hyopneumoniae* was incubated with the plates as a control. Then HRP-conjugated rabbit anti-bovine IgG, rabbit anti-horse IgG, goat anti-rabbit IgG, goat anti-swine IgG, goat anti-mouse IgG, and rabbit anti-chicken IgG were incubated for 30 min at 37°C. Recombinant P46 was incubated with rabbit anti-rP46 polyclonal serum and HRP-conjugated goat anti-rabbit IgG. Then after washing in PBST, substrate 3, 3′, 5, 5′- tetramethylbenzidine (TMB) was added to each well for visualizing at 37°C. Reactions were stopped by the addition of 2 M sulfuric acid to each well. The optical density was read at 450 nm in a micro plate reader (Bio-Rad, Hercules, CA, USA).

### Analyzing the Potential IgG-Binding Proteins and IgG-Cleaving Proteins in *Mycoplasma bovis* Strain PG45

In order to identify the potential IgG-binding proteins and IgG-cleaving proteins in *M. bovis* strain PG45, BLASTp search software was used to find the homologous proteins of MIB and MIP. The amino acid sequences of *MMCAP2_0582* (Protein ID: ACU78687.1), *MMCAP2_0583* (Protein ID: ACU78312.1), *MBOVPG45_0375* (Protein ID: ADR24856.1) and *MBOVPG45_0376* (Protein ID: ADR24916.1) were acquired from the National Center for Biotechnology Information. Clustalx and BoxShade softwares were used to analyze the homology of MIB and MBOVPG45_0375, and the homology of MIP and MBOVPG45_0376. SWISS-MODEL software was used to predict the structure and analyze the functional domain of MBOVPG45_0375.

### Expression and Purification of r0375

According to the complete genome sequence of *M. bovis* strain PG45 (23). Nine TGA codons exist in the coding gene sequence of MBOVPG45_0375. TGA is a universal termination codon but it encodes tryptophan in mycoplasmas. When cloning a mycoplasma gene in the *E. coli* expression system, the presence of TGA codon can lead to the early termination of gene translation. The intact open reading frame of the gene sequence of MBOVPG45_0375, stop codon-included and TGA-corrected, was synthesized by Sangon Biotech (Shanghai, China). Expression was performed as previously described (24). Briefly, the gene was digested (*Bam*H I and *Xho* I) and ligated into the pET-28a plasmid to produce pET-MBOVPG45_0375. The plasmid was transformed into *E. coli* BL21(DE3) competent cells. *Escherichia coli* BL21(DE3)/pET-28a-MBOVPG45_0375 transformants were cultivated for 6 h at 37°C with constant shaking (200 r/min), followed by induction using 1 mM isopropyl-β-D-thiogalactoside (IPTG) for 8 h. Cells were collected and disrupted by sonication. The supernatant was separated by centrifugation and the precipitate was re-suspended in phosphate buffer saline (PBS, 10 mM, PH 7.2). Purification was performed using Ni-Affinity Chromatography (Qiagen, NY, USA) following previously described protocols (24), and the solubility and purity of r0375 were analyzed by SDS-PAGE.

### Confirming the Function of r0375 Binding to IgG

The analysis was performed by nickel agarose pull-down assays to assess the interactions between r0375 and IgG. Nickel agarose conjugated with r0375 was incubated with IgG from different species for 1 h at room temperature. Nickel agarose conjugated with r0375 and nickel agarose incubated with IgG were used as controls. Unbound proteins were washed away with PBST. After mixing with protein loading buffer (TransGen Biotech, Beijing, China) and boiling for 10 min, the samples were analyzed by SDS-PAGE.

Western blotting was performed as previously described (25). Briefly, r0375 and recombinant P48 (rP48, Laboratory collection) of *M. bovis* were separated by 12% SDS-PAGE and transferred onto PVDF membrane. After blocking with 5% skim milk in PBST (wt/vol) for 2 h at 37°C, the membrane was incubated with HRP-conjugated IgG from goat, and mouse, respectively, for 1 h at 37°C. Unbound IgG was washed away with 3 × 20 ml of PBST. IgG-coupled peroxidase activity was detected using High-sig ECL Western Blotting Substrate (Tanon Science and Technology, Shanghai, China) according to the manufacturer's indications. Membrane imaging was visualized on a Tanon-5200 Chemiluminescent Imaging System (Tanon Science and Technology).

ELISA plates were coated with bovine, goat, or rabbit IgG (50 μg/ml, 100 μl per well) and incubated overnight at 4°C. PBS and BSA were coated as controls. The plate was then washed and blocked as described above. The different concentrations of r0375 were incubated for 1 h at 37°C. After washing, the primary antibody [Mouse anti-His tag mAb (ABclonal)] and the second antibody (HRP-conjugated goat anti-mouse IgG) were incubated, and the optical density was measured.

### Identifying the Core Region of IgG That Binds r0375

The binding site of IgG was identified using SDS-PAGE. Briefly, Fab or Fc fragments of human IgG were incubated with nickel agarose, or incubated with nickel agarose after pre-incubation with r0375 for 30 min. Unbound proteins were washed away with PBS. These samples were analyzed and stained with coomassie brilliant blue.

The procedure of western blotting is as follows. Light chains and heavy chains of bovine IgG were separated by SDS-PAGE, then transferred onto PVDF membranes and incubated with PBS or r0375 for 30 min at 37°C. After washing with PBST, the primary antibody (Mouse anti-His tag mAb, 1:1,000) and the second antibody (HRP goat anti-mouse IgG, 1:10,000) were incubated. Peroxidase activity was detected as described above.

### Identifying the Functional Region of MBOVPG45-0375 That Binds IgG

Potential functional domains of MBOVPG45_0375 were predicted with SWISS-MODEL software. The partial amino acid sequence from 299 to 615 of MBOVPG45-0375 was amplified. The recombinant MBOVPG45_0375 truncated domain (r0375 TD) was expressed in *E. coli* as described above. The ability of r0375 TD to bind IgG was tested by western blotting. Pre-induced and induced recombinant bacterial lysates were separated by 12% SDS-PAGE and transferred onto PVDF membranes. After blocking, HRP-conjugated goat IgG was incubated for 30 min at 37°C and the peroxidase activity was detected.

### ELISA Assay to Identify Whether r0375 Blocks the Union of Antigen-Antibody

Binding of anti-recombinant phase-variant protein A (rPVPA) of *Mycoplasma galliscepticum* polyclonal serum (Laboratory collection) from chicken to its antigen rPVPA (Laboratory collection), anti-*M. bovis* strain PD polyclonal serum (Laboratory collection) from cattle to the whole bacterial proteins of *M. bovis* strain PD, and anti-*M. bovis* strain PD polyclonal serum from cattle to the antigen rP48 were evaluated, respectively, by using ELISA after pre-incubating with r0375. Binding of the IgG to the rPVPA, or the whole proteins of *M. bovis* strain PD and rP48 in the absence of r0375 were used as controls. HRP conjugated rabbit anti-chicken IgG or HRP conjugated rabbit anti-cattle IgG was incubated and the activity of peroxidase was detected.

### Western Blotting to Identify Whether r0375 Influences the Deposition of IgG and C1q on *Mycoplasma bovis* Strain PD

*Mycoplasma bovis* strain PD was incubated with its polyclonal serum after pre-incubation with r0375, or polyclonal serum alone, or PBS in human negative serum (HNS) (Biolab, Beijing, China) or in absence of HNS, respectively. Proteins that non-specifically bound were washed away with PBS. IgG and C1q that deposited on the surface of *M. bovis* strain PD were detected by western blotting. P48 was used as reference to denote the amount of the bacterium. Gel Image System software was used to analyze the gray-scale value. The ratio of the amount of IgG or C1q fractions to the amount of P48 fraction was used to analyze the relative amount of IgG and C1q.

### Expression, Purification, and Function of r0376

The intact open reading frame of the gene sequence of MBOVPG45_0376, stop codon-included and TGA-corrected, was synthesized by Sangon Biotech (Shanghai, China). The expression and purification of r0376 were performed as described above for r0375. SDS-PAGE and western blotting were used to identify the IgG-cleaving activity of r0376. The same concentration of bovine IgG was incubated with r0375, or r0376, or r0375, and r0376, respectively. In the SDS-PAGE assay, cleavage of IgG from different animal species was stained with coomassie brilliant blue. In the western blotting assay, the HRP-conjugated rabbit anti-bovine IgG was used to detect the IgG heavy chain.

The cleavage activity of r0376 was detected under different conditions, such as incubation time (1, 5, 10, 30, 60, 120, 240 min) and temperature (5, 20, 35, 50, 65, 80°C). IgG incubated with r0375 or r0376 was used as controls. IgG incubated with r0375 and r0376 was used as experimental group. Cleavage of IgG was detected by SDS-PAGE and stained with coomassie brilliant blue.

### Preparation of Rabbit Anti-r0375 and Anti-r0376 Polyclonal Serum

New Zealand White Rabbits were subcutaneous immunized with r0375 or r0376 protein, respectively. The protein was mixed with an equal volume of Freund's complete adjuvant (Sigma, St Louis, MO, USA) for the first immunization or Freund's incomplete adjuvant (Sigma) for subsequent immunization. After three independent immunizations, the serum was collected and purified as previously described (25).

### Subcellular Localization of MBOVPG45_0375 and MBOVPG45_0376 in *Mycoplasma bovis*

Membrane and cytoplasmic proteins of *M. bovis* strain PG45 were fractionated using a Membrane and Cytosol Protein Extraction Kit (Beyotime, Wuhan, China). Briefly, 200 ml of *M. bovis* strain PG45 in mid-log phase was harvested by centrifugation at 12,000 r/min for 30 min. Cell pellets were washed three times with PBS prior to resuspension in 1 mL of membrane protein extraction reagent and completion of the specific extraction steps following the manufacturer's instructions. The concentration of each protein was determined with the BCA Protein Assay Kit. The same amount of whole, membrane and cytoplasmic proteins (5 μg) were separated by 12% SDS-PAGE and transferred onto PVDF membranes for analysis. Then the membrane was saturated with blocking buffer. Primary antibody (Rabbit anti-r0375 or anti-r0376 polyclonal antibodies, 1:1,000) was incubated with the membrane for 1 h at 37°C. Unbound primary antibody was removed by washing in PBST. The secondary antibody (HRP-conjugated goat anti-rabbit IgG, 1:10,000) was incubated for 30 min at 25°C. After washing with PBST, antibody-coupled peroxidase activity was detected as described above. The monoclonal antibodies against an invariable lipoprotein exposed on the membrane named P48 and the polyclonal serum against a cytoplasmic protein named purine-nucleoside phosphorylase (PNP) were used to appraise the fractionation of membrane and cytoplasmic proteins (22, 25). Gel Image System software was used to analysis the gray-scale value. Ratio of the protein amount in membrane or cytoplasmic fractions to the total protein in whole-cell lysate was used to assess the subcellular localization of MBOVPG45_0375 and MBOVPG45_0376.

### Statistical Analysis

GraphPad Prism 6 (GraphPad Software) was used for graph design and statistical analyses. Statistical analysis was done using a ratio-paired two-tailed *t*-test. All experiments were carried out in triplicate, and data are given as means ± standard deviations (SD).

## Results

### *Mycoplasma bovis* Strain PG45 Showed IgG-Binding Activities

**Figure 1:**
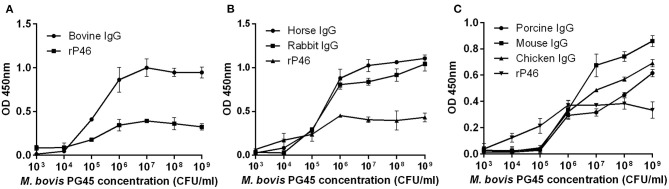
ELISA assessment of the *M. bovis* strain PG45 interaction with IgG. The different concentrations of *M. bovis* strain PG45 were coated on ELISA plates, and the same concentration of IgG and HRP conjugated antibody were incubated successively. Recombinant P46 was incubated as a control. **(A)** ELISA plates incubated with bovine IgG. **(B)** ELISA plates incubated with horse and rabbit IgG. **(C)** ELISA plates incubated with porcine, mouse and chicken IgG. Peroxidase activity was measured using TMB substrate. Error bars correspond to the SD of three technical replicates.

*Mycoplasma bovis* can bind IgG molecules from cattle, horse, rabbit, swine, mouse, and chicken. In the initial stage, as the concentration of *M. bovis* increases, the optical density (OD) value gradually increased. However, for bovine, horse and rabbit IgG, when the concentration of bacteria is relatively high, the OD value increases slowly or even stopped increasing (Figure 1). This result may be due to the saturation of IgG binding. The result confirms that one or more immunoglobulin G-binding proteins exist in *M. bovis*.

### Analysis of Potential IgG-Binding Proteins and IgG-Cleaving Proteins in *Mycoplasma bovis* Strain PG45

The MIB homologs in *M. bovis* strain PG45 includes MBOVPG45_0372, 0374, and 0375. The MIP homologs in *M. bovis* strain PG45 includes MBOVPG45_0373, and 0376. MBOVPG45_0375 and MBOVPG45_0376 were selected and aligned with MIB and MIP. Amino acid sequence analysis showed that there is 47.04% identity between MIB and MBOVPG45_0375 (Supplementary Figure 1A), and 40.77% identity between MIP and MBOVPG45_0376 (Supplementary Figure 1B). The predicted structure of MBOVPG45_0375 (Supplementary Figure 2A) has similarity with the structure of protein M in *M. genitalium*, despite there being no significant amino acid sequence similarity between them.

### Expression and Purification of r0375

The full-length gene of MBOVPG45_0375 was successfully expressed in *E. coli* BL21(DE3) as a His tagged soluble protein. The native MBOVPG45-0375 protein has a predicted molecular size of 89.35 kDa. A slight shift in protein size between r0375 and the native MBOVPG45-0375 resulted from the presence of the N-terminal His-tag and additional vector encoded sequences. In order to obtain a protein with functional activity for the subsequent experiments, soluble r0375 in the supernatant was purified by affinity chromatography (Supplementary Figure 2B).

### r0375 Showed IgG-Binding Activities

**Figure 2:**
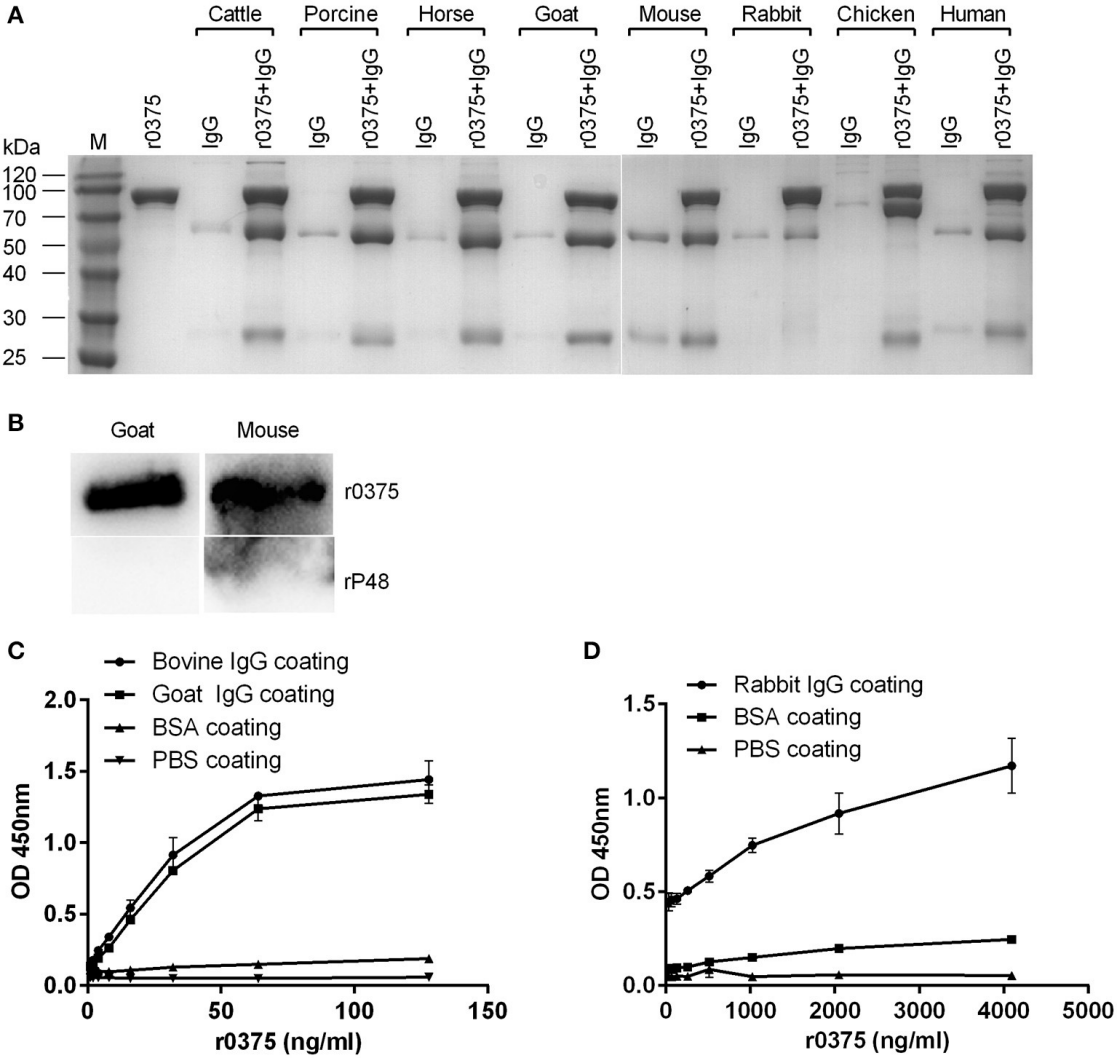
IgG-binding activity of r0375. **(A)** SDS-PAGE identifies the IgG-binding activity of r0375. M, Molecular mass marker. Purified r0375 is conjugated to the nickel agarose *via* its histidine tag, and incubated with non-specific IgG from cattle, swine, horse, goat, mouse, rabbit, chicken, human. After washing, IgG bound to the agarose is detected by SDS-PAGE. The control group includes the nickel agarose conjugated r0375 and the nickel agarose incubated with IgG. **(B)** Western blotting identifies the IgG-binding activity of r0375. r0375 and rP48 were separated by SDS-PAGE and transferred onto PVDF membrane, then HRP-conjugated IgG of goat, or mouse was incubated, respectively. IgG bound to the membrane is detected. **(C)** ELISA identifies the IgG-binding activity of r0375. Bovine or goat IgG (5 μg per well) were coated on the ELISA plates, PBS or BSA (5 μg per well) were coated as controls. The plates were incubated with r0375 at different concentrations. Using mouse anti-His tag IgG as the primary antibody and HRP-conjugated goat anti-mouse IgG as the second antibody. The value of optical density was measured with TMB as substrate. **(D)** Rabbit IgG (5 μg per well) was coated on ELISA plates and other procedures are the same as described in **(C)**. Error bars correspond to the SD of three technical replicates.

The IgG-binding activity of r0375 was assessed using different experimental methods. In the SDS-PAGE assay, r0375 can bind to IgG derived from cattle, swine, horse, sheep, mouse, rabbit, chicken and human (Figure 2A). Although r0375 has relatively weak affinity for IgG of rabbit. There is a slight non-specific binding between IgG and nickel agarose, especially for mouse IgG. Through ELISA and western blotting, it is further confirmed that r0375 can indeed bind to IgG non-immunologically (Figures 2B–D). In summary, this result reveals that r0375 is an IgG-binding protein of *M. bovis*, and probably has similar functions to MIB from *M*. subsp. *capri* and protein M from *M. genitalium*.

### The Binding Region of IgG and r0375

**Figure 3:**
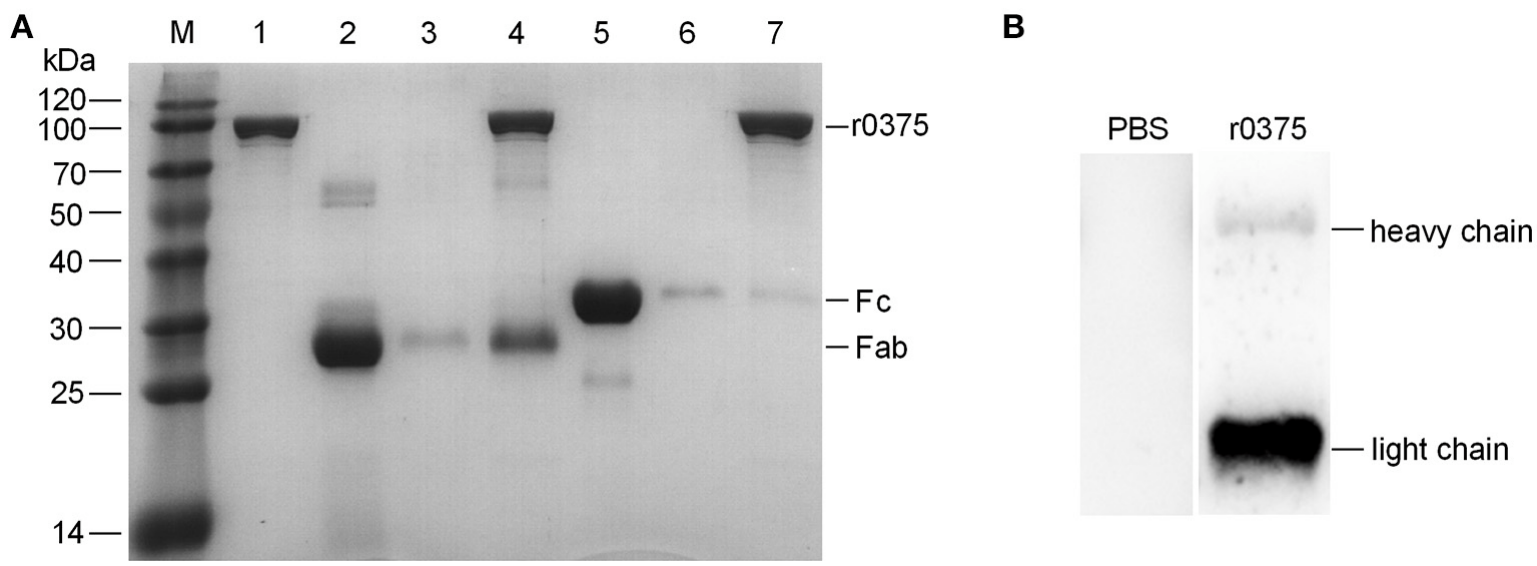
The binding region of IgG and r0375. **(A)** SDS-PAGE identifies that r0375 binds to the Fab fragment of human IgG. M, Molecular mass marker; Lane 1, Nickel agarose conjugated r0375; Lane 2, Fab fragment; Lane 3, Nickel agarose incubated with the Fab fragment; Lane 4, Nickel agarose conjugated r0375 incubated with the Fab fragment; Lane 5, Fc fragment; Lane 6, Nickel agarose incubated with the Fc fragment; Lane 7, Nickel agarose conjugated r0375 incubated with the Fc fragment. **(B)** Western blotting identifies that r0375 can bind to the light chain of IgG. Light chains and heavy chains of bovine IgG were separated by SDS-PAGE, transferred to PVDF and then incubated with PBS or r0375. The binding position was detected with mouse anti-His tag mAb and HRP-conjugated goat anti-mouse IgG.

SDS-PAGE assay indicates that r0375 can bind to the Fab fragment of human IgG but not the Fc fragment (Figure 3A). Western blotting indicates that r0375 primarily combined with light chain of IgG (Figure 3B). In order to analyze the functional domain of MBOVPG45-0375 that binds IgG, r0375 TD was expressed in *E. coli* as the predicted key part of the MBOVPG45-0375 (Supplementary Figure 3A). Western blotting confirmed that r0375 TD can bind to bovine IgG (Supplementary Figure 3B).

### r0375 Blocks the Formation of Antigen-Antibody Complex and the Deposition of C1q on *Mycoplasma bovis*

**Figure 4:**
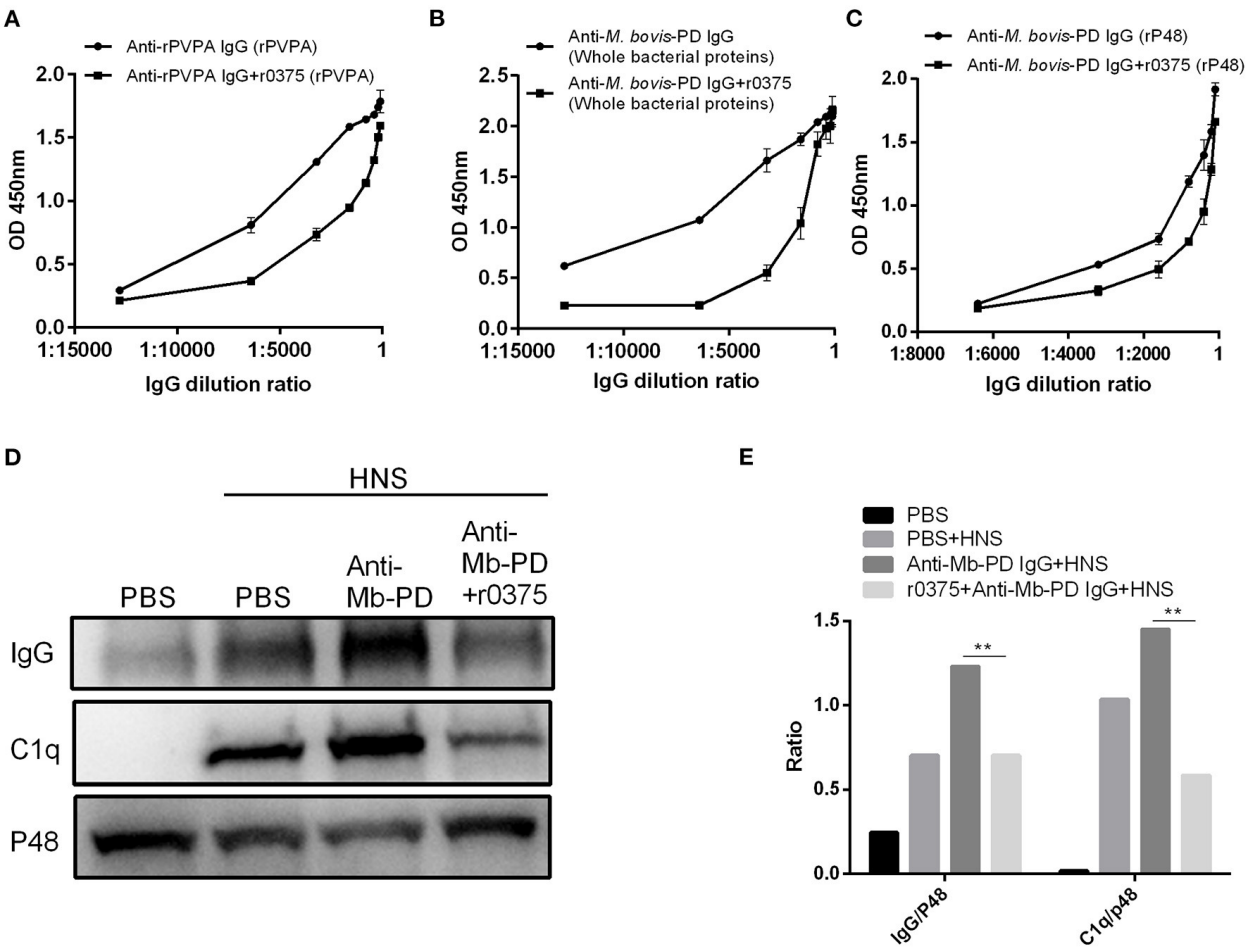
The ability of r0375 to block the union of antigen-antibody and deposition of C1q mediated by IgG. **(A)** Interaction of anti-rPVPA polyclonal serum and its antigen rPVPA was evaluated after pre-incubation of serum with r0375. Anti-rPVPA polyclonal serum combines with rPVPA as a control. **(B)** Interaction of anti-*M. bovis* polyclonal serum and the whole bacterial proteins of *M. bovis* strain PD was evaluated as in **(A)**. **(C)** Interaction of anti-*M. bovis* polyclonal serum and rP48 was evaluated as in **(A)**. Error bars correspond to the SD of three technical replicates. **(D)** Western blotting detects that r0375 inhibit the deposition of IgG and C1q on the bacterial surface. *Mycoplasma bovis* incubated with PBS in HNS or in absence of HNS as controls. The other two groups are *M. bovis* incubated with its polyclonal serum or its polyclonal serum that pre-incubated with r0375. The amount of IgG and C1q on the surface of *M. bovis* was detected, and P48 was used to reflect the number of bacteria. **(E)** Graph showing the ratio of the protein amount in IgG or C1q fraction to the protein amount in p48 fraction. The asterisk above the charts stands for statistically significant differences. **P ≤ 0.01.

The ability of r0375 to block the formation of antigen-antibody complexes was determined. Recombinant 0375 can partially inhibit the interaction of polyclonal serum and its antigen when the serum is pre-incubated with r0375 (Figures 4A–C). Another assay reveals that r0375 can inhibit the deposition of C1q on *M. bovis* by inhibiting the combined quantity of IgG on the surface of the bacteria. As an important component in the classical complement pathway, C1q mainly binds to the Fc portion of IgG. Little deposition of IgG and C1q on *M. bovis* was observed in the absence of anti-*M. bovis* polyclonal serum. After adding the polyclonal serum, the deposition of IgG and C1q on the bacteria increases. Notably the deposition decreases significantly with pre-incubation of polyclonal serum and r0375, because r0375 competitively combines with IgG (Figures 4D,E).

### r0376 Showed IgG-Cleaving Activities

**Figure 5:**
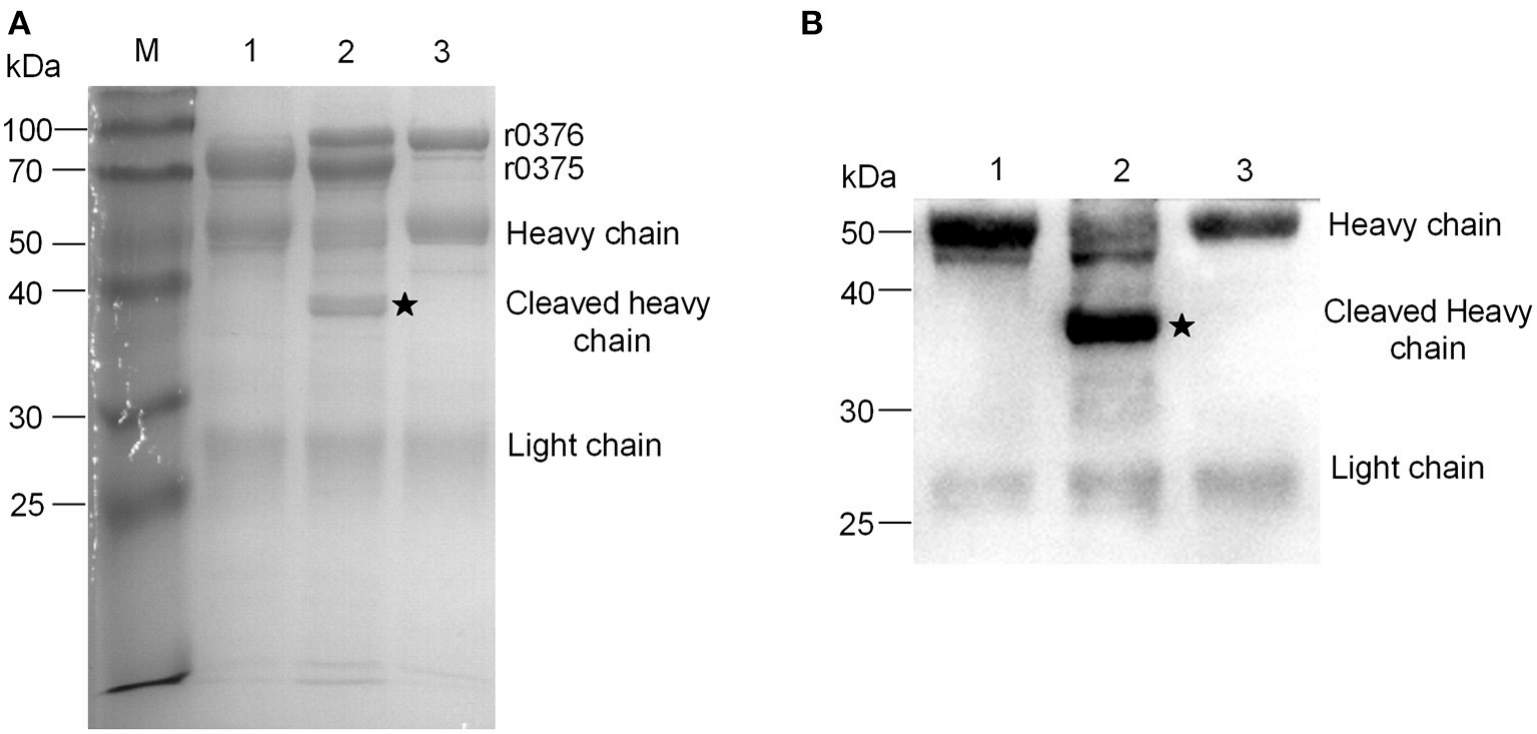
IgG-cleaving activity of r0376. **(A)** SDS-PAGE assay detects the effect of r0376 cleaving bovine IgG. M, Molecular mass marker; Lane 1, bovine IgG incubated with r0375; Lane 2, bovine IgG incubated with r0375 and r0376; Lane 3, bovine IgG incubated with r0376. **(B)** Western blotting detects the full-length or cleaved heavy chain of bovine IgG. Lane 1, bovine IgG incubated with r0375; Lane 2, bovine IgG incubated with r0375 and r0376; Lane 3, bovine IgG incubated with r0376. Black star, cleaved IgG heavy chain fragment.

MBOVPG45_0376 is a putative lipoprotein and one domain of the protein may be a peptidase based on bioinformatic analyses. r0376 was expressed in *E. coli* and purified (Supplementary Figure 4). In comparative analysis of the interaction of r0375, IgG and r0376, we found r0376 can cleave the heavy chain of bovine IgG based on the combination of r0375 and IgG (Figures 5A,B). Furthermore, r0376 can also cleave IgG from swine, horse, goat, and human (Supplementary Figure 5). This reveals that r0376 and MIP of *M. mycoides* subsp. *capri* have sequence similarity and have equivalent functions, with r0376 acting as an IgG-cleaving protein of *M. bovis*.

### Effect of the Incubation Time and Temperature on the Cleavage of IgG

The cleavage activity of r0376 was monitored under different conditions. The fragment of cleaved IgG heavy chain increases with the increasing incubation time (Supplementary Figure 6A), with greatest activity observed around 35°C. This activity was lost above 65°C (Supplementary Figure 6B). These results indicate that r0376 exhibits a protease activity. The conditions needed for its function are relatively mild, which is close to the temperature of the bovine body.

### Subcellular Localization of MBOVPG45_0375 and MBOVPG45_0376 in *Mycoplasma bovis*

**Figure 6:**
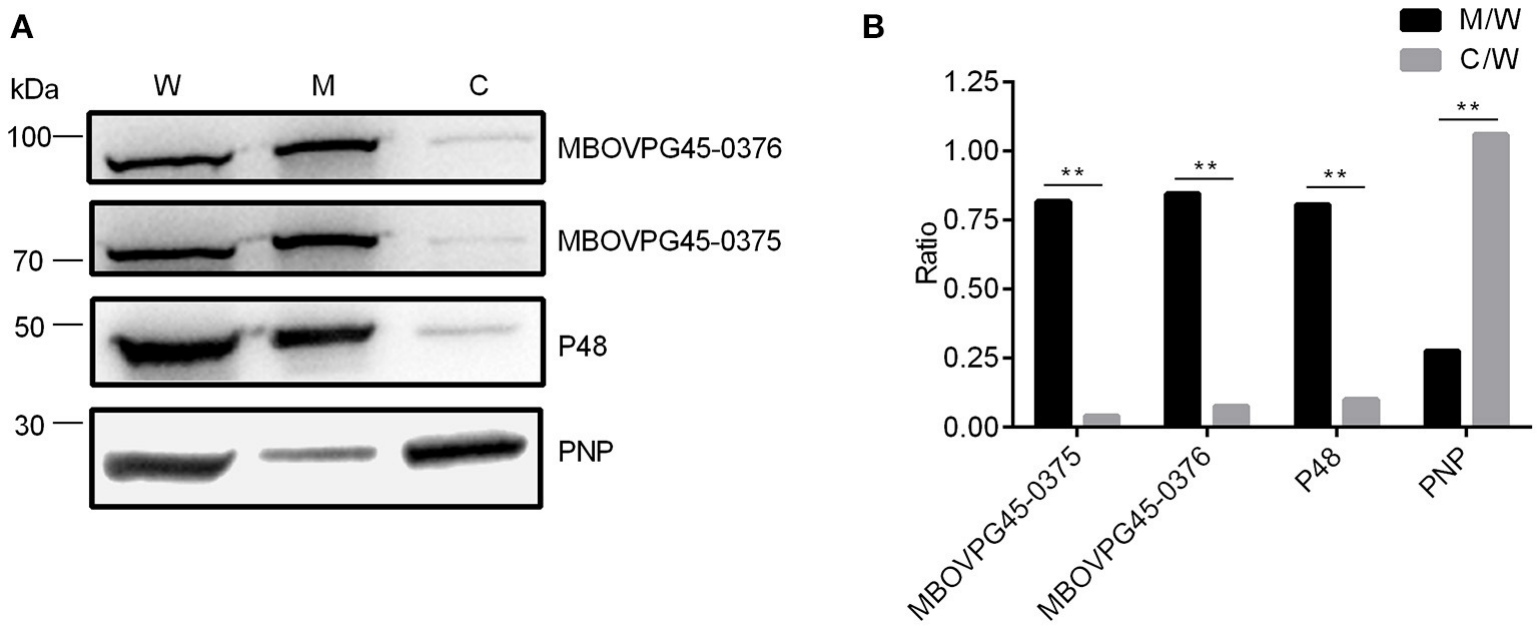
Subcellular localization of MBOVPG45-0375 and MBOVPG45-0376 in *M. bovis*. **(A)** Western blotting analyzes the subcellular localization of MBOVPG45-0375 and MBOVPG45-0376. W, whole proteins of *M. bovis* strain PG45; M, membrane proteins; C, cytoplasmic proteins. **(B)** Graph showing the ratio of the protein amount in the membrane or cytoplasmic fractions to the total proteins in whole-cell lysate. The asterisk above the charts stands for statistically significant differences. **P ≤ 0.01.

To identify the location of the two target proteins on *M. bovis* strain PG45, SDS-PAGE assay was performed on subcellular fractions. The results indicate that MBOVPG45_0375 and MBOVPG45_0376 were detected predominantly in the membrane fraction (Figure 6). The monoclonal antibody (mAb) directed against known membrane protein P48 and the polyclonal serum against known cytoplasmic protein PNP were included as controls.

## Discussion

In this study, we first used ELISA assay to confirm that *M. bovis* strain PG45 contains IBP. Then, MBOVPG45-0375 and MBOVPG45-0376 were chosen to assess the function of MIB and MIP homologs in *M. bovis* strain PG45. There is sequence similarity between MIB and MBOVPG45-0375, and between MIP and MBOVPG45-0376. We identified r0375 as an IgG-binding protein of *M. bovis* and that the binding of r0375 to IgG inhibits the formation of antigen-antibody union. Furthermore, we identified that r0376 is an IgG-cleaving protein of *M. bovis*, and cleaves the heavy chain of IgG. Moreover, MBOVPG45_0375 and MBOVPG45_0376, encoded by two adjacent genes, are membrane proteins of *M. bovis* strain PG45.

MBOVPG45_0375 has a strong amino acid sequence similarity with MIB, and the functions of these two proteins are similar. However, there are some differences between them. Unlike MIB, r0375 is able to bind to purified IgG from rabbit. Moreover, the predicted structure model of MBOVPG45_0375 showed similarity to protein M of *M. genitalium*, despite the few sequence similarities between them. There is also a very strong structural similarity between MIB from *M. mycoides* subsp. *capri* and protein M from *M. genitalium* (14). These three proteins from different mycoplasma species, protein M, MIB, and MBOVPG45_0375, have a common function of binding IgG. It suggests that the minimal bacterium may contain a relatively conserved protein. Although their sequences may not have similarity due to different evolutionary processes in various hosts, their IgG-binding function has not changed. These mycoplasma species may have the function of binding host IgG to form camouflage on its surface for circumventing the recognition of immune cells; or they can bind specific IgG against mycoplasma for blocking the immunological interaction of antigen-antibody, and inhibiting the host's killing effect on *M. bovis* mediated by antibodies.

In the process of confirming r0375 binding to IgG non-immunologically, we found r0375 has a different affinity to different types of IgG from human and animals. More specifically the affinity of r0375 binding to IgG from bovine and goat is higher than IgG from ribbit. A similar characteristic that has a different affinity to different types of IgG was also found with protein M (13). Several IBP that have different structures and functions may simultaneously exist in a bacterial strain. For example, in *Streptococcus pyogenes*, the structure between Sib35 and M12 proteins have no homology, and their function is different. M12 protein interacts with human IgG3 (26), but Sib35 reacts with all four human IgG subclasses (27). Besides MBOVPG45-0375 and its paralogs, whether *M. bovis* contains other IBP that different from the homologs of MIB is unclear.

The union of antigen and antibody depends on the formation of the spatial complementary structure. The six complementarity determining regions (CDRs) of variable regions of IgG participate in antigen recognition (28, 29). Despite preferential binding to the light chain, the combination of r0375 and IgG may occupy the antigen recognition site of IgG and block the formation of antigen-antibody complexes. In the process of antigen recognition, the heavy chain plays a more important role than the light chain (30). Thus, after r0376 cleavage, an IgG with incomplete heavy chain basically loses the function of recognizing its antigen. More importantly, the binding and cleaving activities of 0375 and 0376 are non-specific. In conclusion, the synergistic activities between r0375 and r0376 indicates these proteins could be a virulence factor that limits the host humoral immunity.

The strong similarity of the location of genes and the function of proteins indicates MBOVPG45-0375 and MBOVPG45-0376 have comparability with MIB and MIP. This similarity has confirmed the prediction by Arfi et al. that MIB-MIP system homologs are widely distributed in the majority of human and animal pathogenic Mycoplasma. Similar to MIB / MIP, the proteolytic activity of r0376 is not functional when IgG interacts with r0376 alone, as r0375 is required for its activity. Whether r0375 binds to IgG and changes the spatial conformation of IgG so that r0376 can play the activity of cleaving IgG is still unknown. Therefore, the structure of r0375, r0376, binary r0375: IgG complexes, or tripartite r0375: IgG: r0376 complexes need further analysis to clarify the interaction mechanism.

The membrane proteins of bacteria usually perform very important roles in bacterial infections, being involved in multiple biological processes i.e., cytoadherence, nutritional access, and immune evasion (31, 32). Most known IBP are located on the bacterial surface. It is worth noting that some IBP have more than one function. For instance, the *S. aureus* protein Sbi can bind IgG, complement C3 and Factor H (FH), which forms tripartite Sbi: C3b: FH complexes that can interfere with innate immune recognition (33). The M protein of *S. pyogenes* is another multifunctional bacterial protein, which can bind the complement regulators FH, Factor H like protein 1, C4BP (C4b binding protein), and other plasma proteins, i.e., plasminogen, fibronectin, thrombin, fibrinogen, IgA, IgG and kininogen (26, 34–37). Thus, as the membrane protein of *M. bovis* strain PG45, MBOVPG45-0375 may have other functions besides binding to IgG, and the possibility of interacting with other substances needs further research.

This study on the IgG-binding protein and IgG-cleaving protein of *M. bovis* suggests that the bacterial pathogen may use complex mechanisms to proliferate in the bovine respiratory tract as well as other sites, causing multiple chronic diseases. Although we have confirmed that r0375 and r0376 could bind and cleave IgG *in vitro*, further studies on how it works in the natural microenvironment and whether the microorganism would exploit this mechanism to evade the host immunity need investigation. It is also an interesting question of whether the expression level of MBOVPG45-0375 and MBOVPG45-0376 on the bacterial membrane is influenced by quantity and type of IgG in the bacterial growth environment. In summary, this finding of IgG-binding protein and IgG-cleaving protein in *M. bovis* will help us to understand how diminutive pathogens fight with powerful host immunity.

## Data Availability Statement

The original contributions generated in the study are included in the article/Supplementary Material, further inquiries can be directed to the corresponding author.

## Ethics Statement

The animal study was reviewed and approved by Beijing Administration Committee of Laboratory Animals.

## Author Contributions

WW, CP, and YZ conceived the idea of the project. HZ, YZ, and ZW designed the experiments. HZ and PW performed the experiments. CP, HZ, and YZ analyzed the results. WW, HZ, and ML wrote and edited the manuscript. All authors contributed to the article and approved the submitted version.

## Conflict of Interest

The authors declare that the research was conducted in the absence of any commercial or financial relationships that could be construed as a potential conflict of interest.
