# Supplementary material for: MBOVPG45_0375 Encodes an IgG-Binding Protein and MBOVPG45_0376 Encodes an IgG-Cleaving Protein in Mycoplasma bovis
Source: Front Vet Sci. 2021 Apr 15;8:644224. doi: 10.3389/fvets.2021.644224 (PMC8081823; doi:10.3389/fvets.2021.644224)
Supplement: Supplementary file 1 [file Data_Sheet_1.docx]

Supplementary Material

# Homology analysis of MIB and MBOVPG45_0375, MIP and MBOVPG45_0376.

A


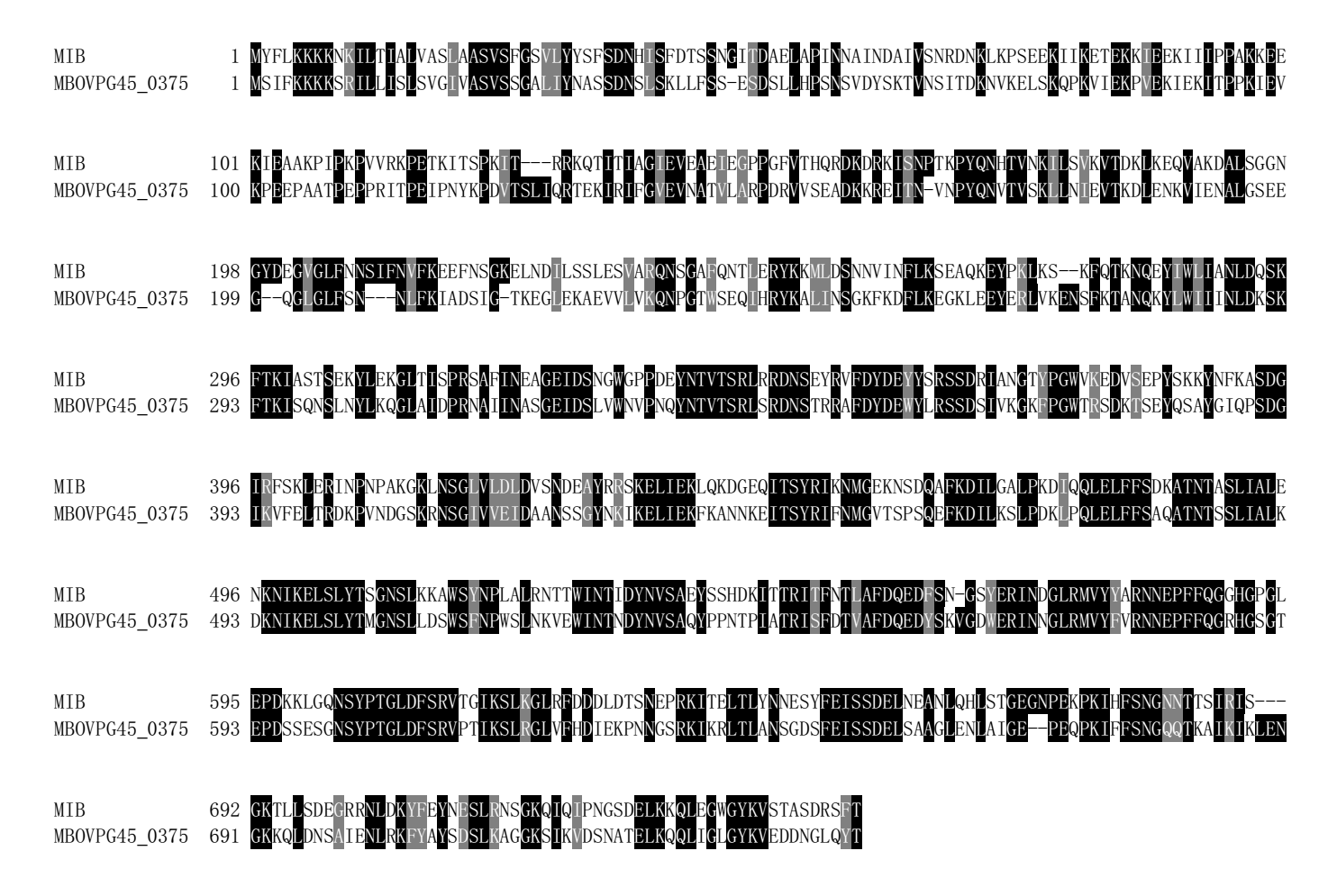


B


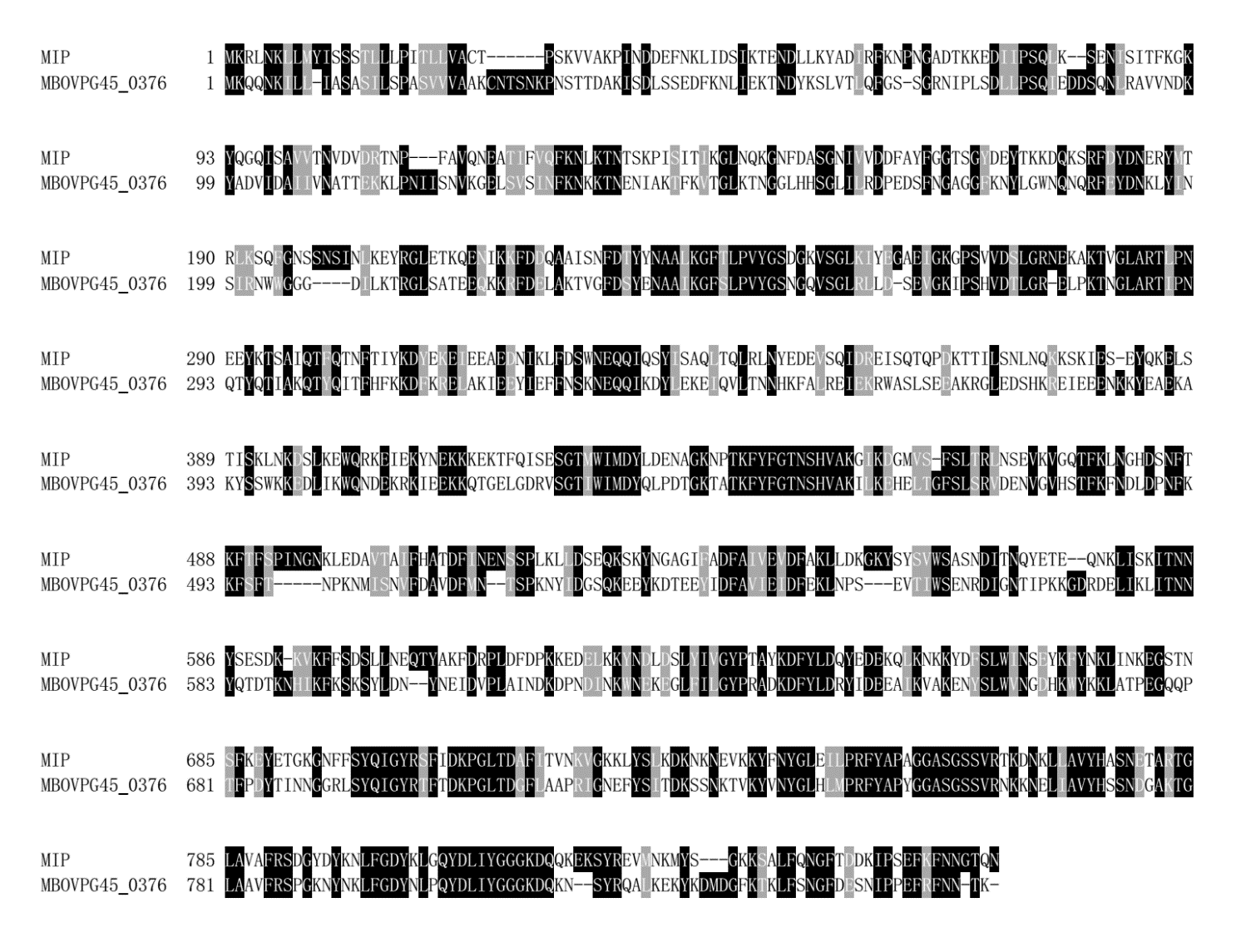


**Supplementary Figure 1.** Homology analysis between MIB and MBOVPG45_0375 (A), MIP and MBOVPG45_0376 (B). The amino acid sequences were aligned using Clustalx software with default parameters. The alignment file was then processed using BoxShade software to generate the figure. Gaps are indicated as bands (“-”), fully conserved residues are highlighted in black, and relatively conserved residues are highlighted in dark grey.

# Structure prediction and recombinant expression of MBOVPG45_0375.


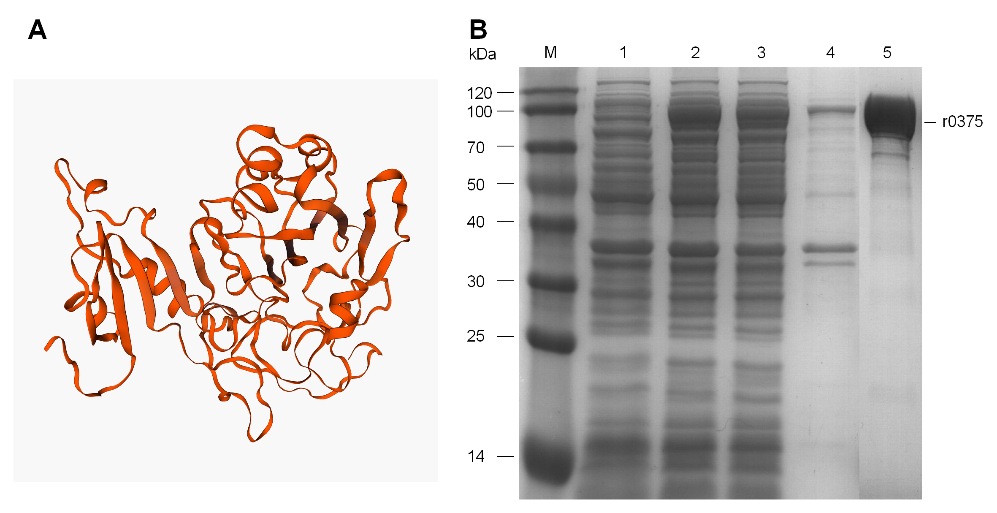


**Supplementary Figure 2.** Structure prediction and recombinant expression of MBOVPG45_0375. (A) Predicted structure model of MBOVPG45_0375 using SWISS-MODEL software. (B) The expression and purification of MBOVPG45_0375. SDS-PAGE analysis of the expression of r0375 in *E. coli* BL21 (DE3). M: Molecular mass marker; Lane 1: Pre-induced bacterial lysate; Lane 2: Induced bacterial lysate; Lane 3: Supernatant of total cell lysate after induction; Lane 4: Precipitate of total cell lysate after induction; Lane 5: Purified r0375 using affinity chromatography.

# The functional region of MBOVPG45_0375 that binds to IgG.


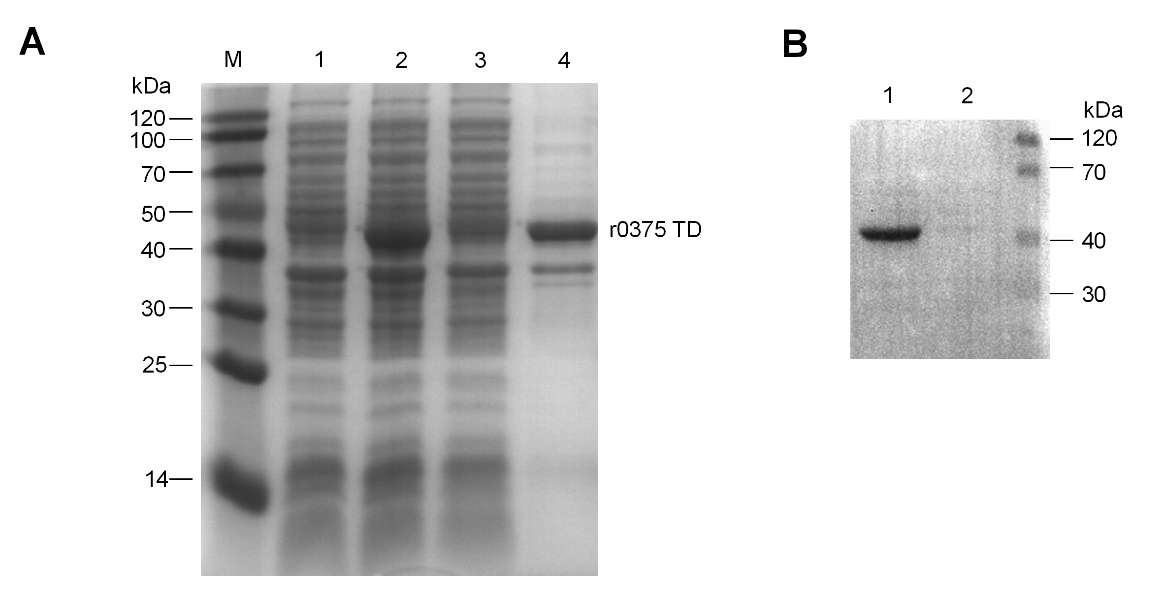


**Supplementary Figure 3.** Expression and function of r0375 TD. (A) SDS-PAGE analysis of the expression of r0375 TD in *E. coli* BL21 (DE3). M: Molecular mass marker; Lane 1: Pre-induced bacterial lysate; Lane 2: Induced bacterial lysate; Lane 3: Supernatant of total cell lysate after induction; Lane 4: Precipitate of total cell lysate after induction. (B) Western blotting identifies that r0375 TD can bind to IgG. lane 1: induced bacterial lysate of r0375 TD; Lane 2: pre-induced bacterial lysate of r0375 TD. Bacterial lysate was separated by SDS-PAGE, then incubated with HRP-conjugated Goat IgG and the activity of peroxidase was detected.

# Recombinant expression of MBOVPG45_0376.


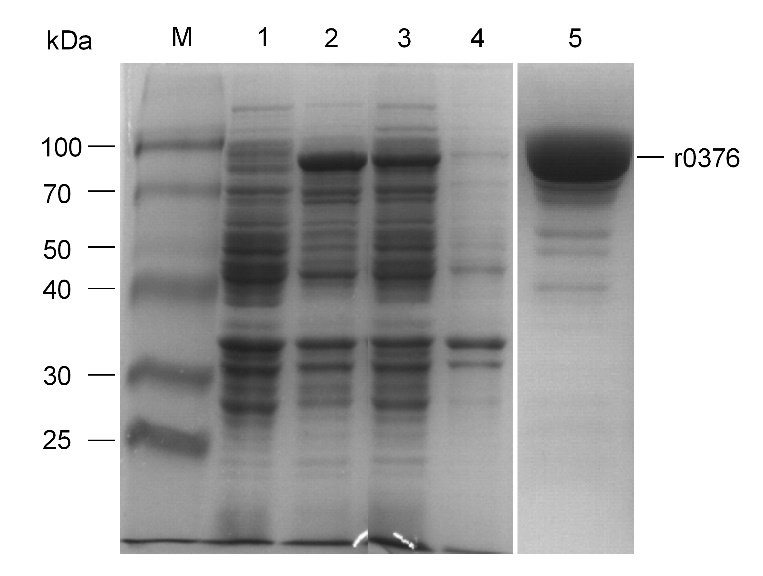


**Supplementary Figure 4.** Expression in *E. coli* BL21 (DE3) and purification of r0376. M: Molecular mass marker; Lane 1: Pre-induced bacterial lysate; Lane 2: Induced bacterial lysate; Lane 3: Supernatant of total cell lysate after induction; Lane 4: Precipitate of total cell lysate after induction; Lane 5: Purified r0376 using affinity chromatography.

# IgG-cleaving activity of r0376.


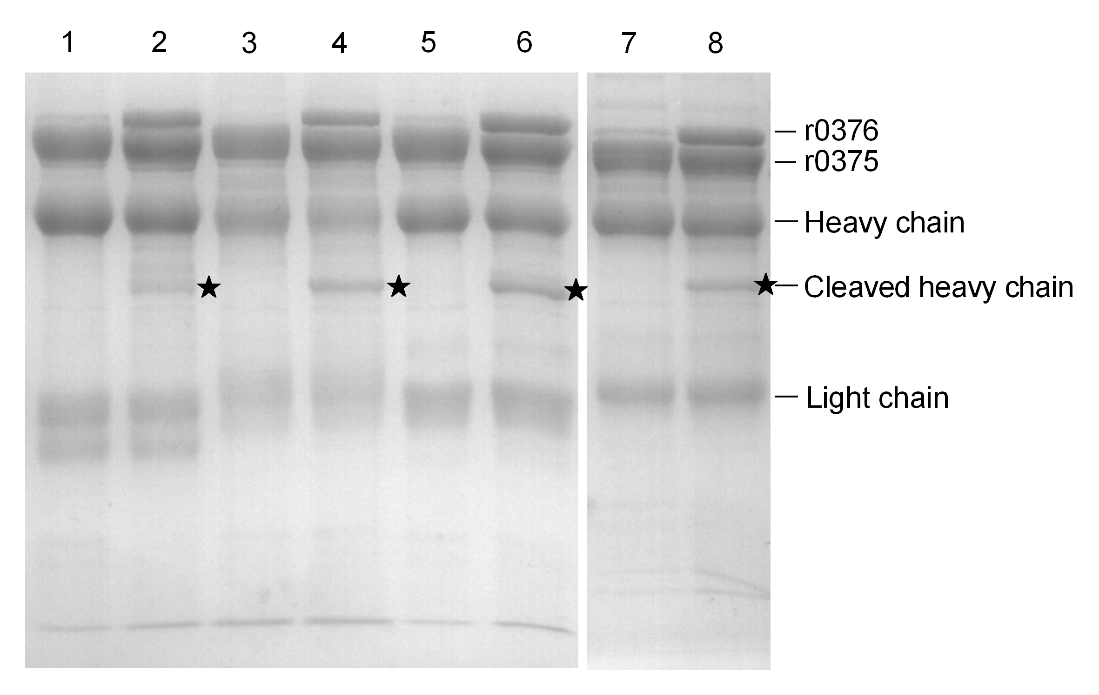


**Supplementary Figure 5.** IgG-cleaving activity of r0376. SDS-PAGE assay detects the effect of r0376 cleaving IgG from several animal species. Lane 1: porcine IgG incubated with r0375; Lane 2: porcine IgG incubated with r0375 and r0376; Lane 3: horse IgG incubated with r0375; Lane 4: horse IgG incubated with r0375 and r0376; Lane 5: goat IgG incubated with r0375; Lane 6: goat IgG incubated with r0375 and r0376; Lane 7: human IgG incubated with r0375; Lane 8: human IgG incubated with r0375 and r0376. Black star, cleaved IgG heavy chain fragment.

# Effect of incubation time and temperature on the lysis of IgG.

**
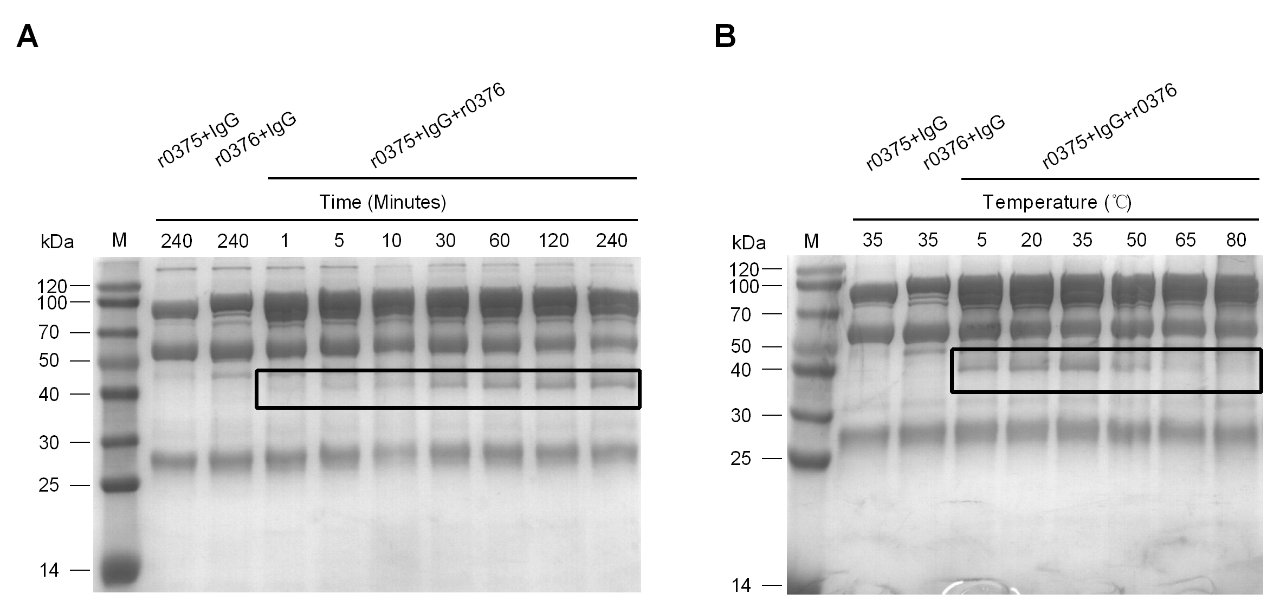
**

**Supplementary Figure 6.** Effect of incubation time and temperature on the lysis of IgG. (A) Effect of incubation time on the lysis of bovine IgG. M: Molecular mass marker. Lane 1: r0375 incubated with IgG for 240 mins; Lane 2: r0376 incubated with IgG for 240 mins; Lane 3 to Lane 9: r0375 incubated with IgG and r0376 for different time. (B) Effect of temperature on the lysis of bovine IgG. M: Molecular mass marker. Lane 1: r0375 incubated with IgG at 35℃; Lane 2: r0376 incubated with IgG at 35℃; Lane 3 to Lane 8: r0375 incubated with IgG and r0376 at different temperature. Black rectangle, cleaved IgG heavy chain fragment.
